# Accompanying Titanium Meshes and Titanium-Reinforced Membranes with Collagen Membranes in Vertical Alveolar Ridge Augmentations: A Systematic Review

**DOI:** 10.3390/jfb16070246

**Published:** 2025-07-04

**Authors:** Amir-Ali Yousefi-Koma, Reza Amid, Anahita Moscowchi, Hanieh Nokhbatolfoghahaei, Mahdi Kadkhodazadeh

**Affiliations:** 1 Dentofacial Deformities Research Center, Research Institute of Dental Sciences, Shahid Beheshti University of Medical Sciences, Tehran 19839-63113, Iran or amiraliykoma@sbmu.ac.ir (A.-A.Y.-K.); or amidr@sbmu.ac.ir (R.A.); 2 Department of Periodontics, School of Dentistry, Shahid Beheshti University of Medical Sciences, Tehran 19839-63113, Iran; 3 Dental Research Center, Research Institute of Dental Sciences, Shahid Beheshti University of Medical Sciences, Tehran 19839-63113, Iran; a.moscowchi@gmail.com

**Keywords:** alveolar ridge, vertical ridge augmentation, dental implants, guided bone regeneration, bone augmentation, collagen membrane, titanium mesh, titanium-reinforced membrane, dense polytetrafluoroethylene (dPTFE)

## Abstract

**Background:** Vertical ridge augmentations (VRAs), including guided bone regeneration (GBR) techniques, have been utilized in the reconstruction of deficient alveolar ridges for quite some time. GBR-based VRA procedures are technique-sensitive, operator-dependent, and often lead to complications detected during or after the treatment. The main objective of this systematic review was to include randomized and non-randomized human studies that investigated the regenerative outcome differences, as well as the incidence rates of healing and surgical complications of titanium meshes and/or titanium-reinforced membranes with and without collagen membranes utilized in GBR-based VRA. **Methods:** This systematic review has been prepared and organized according to the preferred reporting items for systematic reviews and meta-analyses (PRISMA) 2020 guidelines and is registered at PROSPERO (Registration ID: CRD420251002615). Medline via PubMed, Scopus, Web of Science, Embase, and the Cochrane Library were searched for eligible studies up to 5 June 2025. Randomized and non-randomized human clinical studies, except for case reports, focused on applying titanium meshes or titanium-reinforced membranes with or without collagen membranes in GBR-based VRA, were eligible. **Results:** A total of 119 patients from three human randomized clinical trials (RCTs) and one case series reported across nine articles were included. The addition of collagen membranes causes no significant differences in vertical bone gain or surgical/healing complication rates. **Conclusions:** The addition of collagen membranes on top of titanium meshes and titanium-reinforced membranes might not be necessary in GBR-based VRA. Further human RCTs are required to reach a reliable conclusion.

## 1. Introduction

Tooth loss caused by either trauma or periodontal disease leads to a concomitant horizontal and vertical loss of supporting alveolar bone. Sufficient height and width of alveolar bone are a necessity for triumphant dental implant therapy [1,2]. Vertical ridge augmentations (VRAs), including guided bone regeneration (GBR) techniques, have been utilized in the reconstruction of deficient alveolar ridges for quite some time [3]. GBR-based VRA procedures are technique-sensitive, operator-dependent, and often lead to complications detected during or after the treatment, with an approximate 16% incidence rate for these complications [1,4,5,6]. These procedures require substantial biological endeavors to regenerate critical-sized alveolar defects that lack the support of bony walls in order to stabilize the clot and graft [7]. The delicate adjustments made to these techniques and biomaterials over the past three decades have remarkably reduced the incidence of various complications; however, surgical and healing complications (i.e., soft tissue dehiscence) remain a major concern due to their higher incidence rates compared to other complications and their undeniable negative impact on the duration and success of the VRA treatments [3,7,8,9]. It has been reported that soft tissue complications, including early or late mesh/membrane exposures, have the highest rates of incidence [10].

GBR-based VRA is established upon applying a combination of bone graft materials and barrier membranes. These membranes make the exclusion of different tissues feasible during the healing period. GBR-based VRA aims to use meshes/membranes that can provide and maintain a steady subperiosteal space preceding the bony defects. By performing this, the osteoprogenitor cells are allowed to colonize the region whilst mechanically excluding the epithelial and connective tissue cells that have a much faster migration pace [11]. As crucial as it is to dissect different tissues and cells by applying these membranes, it is also important to note that if the membrane is completely non-perforated and impermeable, it will hinder the vascularization provided by periosteal vessels in the flap covering the defect; hence, the treated area confined by the membrane will need to solely rely on basal bone vasculature for nourishment. Various reports have shown that resorbable membranes are significantly less effective than non-resorbable meshes and membranes in VRAs [12,13,14]. Titanium meshes and newly introduced perforated titanium-reinforced non-resorbable dense polytetrafluoroethylene (dPTFE) membranes are able to enhance the revascularization of the grafted bony materials from the surgical flap’s vascularization network, facilitating the interaction with the periosteum while also supporting the progenitor cells’ influx [15]. However, a major concern with using titanium meshes or perforated titanium-reinforced dPTFE membranes alone is the risk of compromising one of the principles of GBR: the cell-occlusion property [16,17]. That being said, titanium meshes have been used alone for vertical and horizontal ridge augmentations with respective outcomes. It is still not determined whether the presence or lack of cell-occlusion can result in significant differences in the quality and volume of the regenerated alveolar bone.

To the best of the authors’ knowledge, there are no systematic reviews containing randomized and non-randomized human clinical trials that compare the outcomes of titanium meshes and/or titanium-reinforced dPTFE membranes with and without collagen membranes to determine the need for these supplementary collagen membranes. The main objective of this systematic review was to include randomized and non-randomized clinical human studies that investigated the regenerative outcome differences, as well as the incidence rates of healing and surgical complications of titanium meshes and/or perforated titanium-reinforced dPTFE membranes with and without cross-linked or non-cross-linked collagen membranes utilized in GBR-based VRA.

## 2. Materials and Methods

This study has been prepared and organized according to the preferred reporting items for systematic reviews and meta-analyses (PRISMA) 2020 guidelines [18]. This systematic review has been registered at PROSPERO (Registration ID: CRD420251002615). The study question according to the PICO format was as follows: GBR-based alveolar ridge augmentations (I) using titanium-based meshes/membranes with and without collagen membranes (C) in human adult patients suffering from vertical atrophy in their mandible or maxilla, who are candidates for dental implants (P), in order to gain considerable vertical height (O).

### 2.1. Eligibility Criteria

#### 2.1.1. Types of Studies

Human randomized clinical trials (RCTs), controlled clinical trials, case-controls, and case series were eligible. Case reports were excluded.

#### 2.1.2. Population

Human adult patients suffering from anterior or posterior vertical atrophies in the maxilla or mandible. Buccal dehiscence defects were not considered.

#### 2.1.3. Intervention

Vertical alveolar ridge augmentation in the anterior or posterior of the maxilla or mandible using titanium meshes or titanium-based/reinforced membranes with and without non-cross-linked or cross-linked collagen membranes.

#### 2.1.4. Types of Outcome Measures

##### Primary Outcome

Vertical alveolar bone gain.

##### Secondary Outcomes

Surgical and healing complications, bone density, bone quality (i.e., histological evaluations), horizontal bone gain, bone regeneration rate, implant survival rates, and implant stability (i.e., counter torque, insertion torque, etc.).

### 2.2. Information Sources and Search Strategy

An electronic search was executed in Medline via PubMed, Scopus, Web of Science, Embase, and the Cochrane Library to identify eligible studies. The search included articles up to 5 June 2025. The search queries displayed in Table 1 were considered for the electronic search.

### 2.3. Study Selection and Data Collection

Two reviewers (AY and AM) independently screened the titles and abstracts of articles and included/excluded articles based on the mentioned exclusion criteria. Selected articles were then fully read to see if they passed our inclusion criteria. In case of any disagreements, a third reviewer (MK) was consulted. The demographic and methodological details, along with the outcomes from selected studies, were then extracted and tabulated. In case of any conflicts, a third expert (MK) was consulted.

### 2.4. Data Items

The collected items were as follows: (1) authors’ name; (2) year of publication; (3) number of patients; (4) patients’ gender ratio; (5) patients’ mean age and/or age range; (6) alveolar defects (i.e., defect position, defect size from both mesio-distal and bucco-lingual aspects, and maximum amount of vertical bone loss); (7) surgical procedure details; (8) bone grafting materials (e.g., autologous grafts, allografts, xenografts, combinations of different graft materials, etc.); (9) bone grafting techniques; (10) study phases (e.g., initial surgery, removal of meshes and membranes, dental implantation, functional loading, etc.); (11) study groups; (12) dental implants (i.e., material, brand name, number of used implants, etc.); (13) surgical and healing complications; (14) vertical and horizontal bone gain; (15) augmentation rate; (16) bone density and quality; (17) implant survival and stability; (18) pseudo-periosteum thickness; (19) evaluation methods and periods; and (20) short-term and long-term outcomes of alveolar ridge augmentation.

### 2.5. Quality Assessment

The Cochrane risk-of-bias assessment tools for randomized (RoB-2) [19] and non-randomized studies (ROBINS-I) [20] were used for the included studies. Two reviewers (AY and AM) independently evaluated each study using the prefabricated questions of the mentioned quality assessment tool. In case of any heterogeneities in the results, a third expert (MK) was consulted.

### 2.6. Synthesis Methods

Due to the alarming heterogeneities found in the grafting materials, surgical techniques, alveolar defect positions and sizes, and evaluation methods, conducting a meta-analysis was not feasible. Descriptive analysis, along with graphical and narrative synthesis, was performed.

## 3. Results

### 3.1. Study Selection

**Figure 1:**
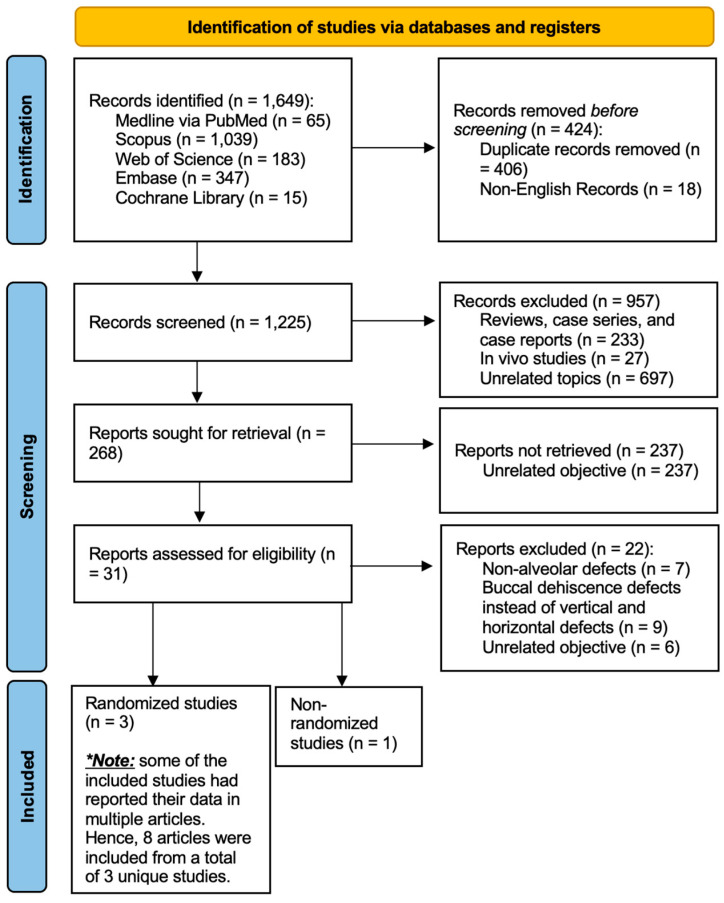
The PRISMA 2020 flow diagram.

During the initial electronic search in the five mentioned databases, a total of 1649 studies were identified. A total of 424 studies, comprising duplicate and non-English records, were removed before the screening process. Out of the 1225 records that were screened, only 31 of them satisfied the eligibility for full-text assessment; the excluded records consisted of human studies that had investigated buccal dehiscence and not vertical or horizontal defects, and overall unrelated topics. Figure 1 details the identification and screening process of this systematic review.

A total of three human RCTs and one case series were included. Two of the included RCTs had published their outcomes in multiple articles due to word count limitations and long-term follow-up sessions. Hence, only four studies were investigated in this systematic review, but a total of nine articles were included in data extraction. The included studies were published between 2013 and 2025; 2013 (n = 1) [21], 2017 (n = 1) [22], 2019 (n = 1) [23], 2021 (n = 2) [24,25], 2023 (n = 1) [26], 2024 (n = 2) [27,28], and 2025 (n = 1) [29]. The included studies came from Italy (n = 7) [22,23,24,25,26,27,28] and the United States of America (n = 1) [29]. Included studies were published in four peer-reviewed journals: Clinical Oral Implants Research (n = 4) [24,25,27,28], Clinical Implant Dentistry and Related Research (n = 3) [22,23,26], Journal of Clinical Periodontology (n = 1) [29], and International Journal of Periodontics and Restorative Dentistry (n = 1) [21] (Supplementary Table S1).

### 3.2. Results of Individual Studies

All of the aforementioned data items extracted from the three included RCTs and one case series are displayed in Supplementary Table S1.

### 3.3. Study Characteristics

#### 3.3.1. Study Design

All of the included studies—three RCTs and one case series—were executed in a standard setting by either a periodontist or an oral surgeon.

#### 3.3.2. Demographics

A total of 119 adult patients were enrolled in this study, with consent forms obtained from all patients in all four included studies. Enrolled patients comprised 47 men and 72 women. One of the studies did not specify the mean age or age range of their patients [24,27,28]. The mean age of the patients was reported in three of the studies as follows: 52 [22,23,25,26], 51.2 ± 10.6 [29], and 50.2 ± 14.4 [21] years old.

#### 3.3.3. Patient Enrollment Exclusion Criteria

All included studies had listed the following exclusion criteria while enrolling the right participants in their studies: poor oral hygiene; untreated previous oral diseases; smoking > 10 cigarettes per day; alcohol and/or drug abuse; pregnancy; presence of local and/or systemic infection; metabolic and/or autoimmune diseases; radiotherapy in the head and neck region in the last 5 years; immune-suppressing or immune-compromising treatments; and bisphosphonate intake.

#### 3.3.4. Surgical Rationale and Procedure

Three-dimensional vertical and/or horizontal ridge augmentation followed by dental implantation was the main objective and aim of all included studies. Perforations in the cortical bone (i.e., decortication) were performed in all three of the included RCTs to promote the migration of osteogenic and osteoprogenitor cells [22,23,24,25,26,27,29]. In all the included studies, bone grafting was accomplished by mixing autogenous bone (harvested from posterior mandible and external oblique ridge) with either xenograft (50:50 autograft and xenograft) (Zcore^®^, Osteogenics Biomedical, or DBBM; Bio-Oss, Geistlich Pharma AG, Wolhusen, Switzerland) [24,27,28,29], or allograft (50:50 autograft and allograft) (EnCore, Osteogenics Biomedical, Lubbock, TX, USA, or BioOss, OsteoHealth, Geistlich, Germany) [21,22,23,25,26]. These bone grafting materials were covered by a variety of combinations of different titanium meshes and titanium-reinforced dPTFE membranes with or without natural or cross-linked collagen membranes in alveolar ridge augmentation. Table 2 showcases the different study groups in the four included studies and the comparisons made between each two study groups. The combination of titanium meshes with cross-linked collagen membranes was the most used method of grafting, with two of the studies following this methodology [22,23,24,25,26,27,28]. Three types of titanium mesh were used in the included studies: (1) CAD/CAM customized titanium meshes [24,27,28]; (2) Pre-shaped titanium meshes on study casts (Jeil Medical, Seoul, Korea), and (3) Pre-fabricated Trinon titanium meshes (Trinon Titanium; Karlsruhe, Germany) [22,23,25,26]. Two of the included studies had used non-resorbable perforated (RPM, Osteogenics Biomedical) [29] or non-perforated (Cytoplast Ti-250XL; Osteogenics Biomedical) dense polytetrafluoroethylene (dPTFE) membranes [22,23,25,26].

#### 3.3.5. Alveolar Defects

The location of the alveolar defects was reported in all four studies. In three of the studies, these defects were not limited to one location and included defects in the anterior and posterior of the mandible and maxilla [21,24,27,28,29], while in one study, the defects were restricted to the posterior mandible [22,23,25,26]. The mean size of the vertical alveolar defects was only reported in three studies, ranging from 3.8 ± 0.7 mm to 10.0 ± 3.8 mm in different study groups [21,22,23,25,26,29]. The mean mesio-distal length of these defects was reported in only one study as 20.07 ± 6.67 mm and 20.07 ± 7.63 mm in its two study groups [29].

#### 3.3.6. Dental Implantation

Two of the included studies did not specify the type or total number of implanted titanium dental implants in their patients [21,29]. The other studies reported 71, 108, and 30 (209 in total) dental implants used for their patients. Two of the studies used the same brand and model of titanium dental implant for all of their cases (i.e., BT Safe^®^, BTK, Biotec Srl, Dueville, Italy) [22,23,24,25,26,27,28].

#### 3.3.7. Study Variables

Table 3 details the study outcomes assessed in the included studies, along with their evaluation methods and periods. Surgical and healing complications were one of the most investigated outcome measures in all included studies. All four of the included studies followed Fontana et al.’s classifications for surgical and healing complications [21,22,23,24,25,26,27,28,29]. Fontana et al. designed the surgical and healing complication classifications specifically for alveolar guided bone regeneration procedures using non-resorbable meshes/membranes [8]. Surgical complications are categorized into three classes: flap damage (i.e., soft tissue perforation or laceration) (Class A); neurological damage (i.e., paresthesia or dysesthesia) (Class B); and vascular damage (i.e., hemorrhage) (Class C). While the healing complications are classified in four tiers: small membrane exposure (≤3 mm) without purulent exudate (Class I); large membrane exposure (>3 mm) without purulent exudate (Class II); membrane exposure with purulent exudate (Class III); and abscess formation without membrane exposure (Class IV).

Two of the included studies investigated the existence and thickness of pseudo-periosteum (i.e., a dense layer of connective tissue with no mineralization and low-cellularity, found under titanium meshes or PTFE membranes) using a UNC-15 periodontal probe [24,27,28,29] following the classification for pseudo-periosteum thickness, established by Cucchi et al. [30]: regular connective tissue with <1 mm in thickness (Type 1); regular connective tissue with 1–2 mm in thickness (Type 2); regular connective tissue with >2 mm in thickness or irregular (i.e., poorly vascularized or non-vascularized) connective tissue in any thickness (Type 3).

### 3.4. Outcomes and Complications

#### 3.4.1. Surgical and Healing Complications

The number of patients that suffered from surgical and/or healing complications in each of the different combinations of meshes and membranes, along with their pseudo-periosteum evaluations, is detailed in Table 4. Out of the 100 patients enrolled in the three included RCTs, 99 of them were evaluated for surgical and healing complications at 6 or 9 months after the bone augmentation procedure. Only one patient did not qualify for the evaluation of surgical and healing complications since they suffered from an accident that damaged multiple oral and maxillofacial regions; this patient belonged to the titanium mesh with cross-linked collagen membrane group [22,23,25,26]. None of the cases in any of the three RCTs suffered from Class C surgical complications (i.e., vascular damage and hemorrhage). Perforated titanium-reinforced dPTFE membranes with and without non-cross-linked collagen membranes did not cause any surgical complications and only resulted in two cases of minor Class 1 healing complications that were resolved. Class III and IV healing complications, which are considered major complications that can directly impact the quality of bone augmentation, were reported in three groups: titanium mesh alone, titanium mesh with cross-linked collagen, and non-perforated titanium-reinforced dPTFE. The combination of titanium mesh with non-cross-linked collagen resulted in no healing complications and only led to one Class A surgical complication that was resolved.

Only two of the studies had evaluated and reported the pseudo-periosteum thickness with a total of 60 patients [24,27,28,29]. Overall, Type 1 pseudo-periosteum was most reported, followed by Type 2 and Type 3. In both studies, the number of Type 2 and Type 3 pseudo-periosteum thicknesses was higher in the groups that did not have any collagen membranes; however, this difference was statistically insignificant. In one study, the number of Type 1 pseudo-periosteum cases was significantly higher in the group that covered the perforated titanium-reinforced dPTFE membranes with non-cross-linked collagen membranes compared to the group that used the dPTFE membranes alone (P = 0.014) [29].

In the included case series, with all patients receiving titanium meshes covered with cross-linked collagen membranes, pseudo-periosteum thickness was not reported. One of the patients suffered from infectious early-term titanium mesh exposure (Class IV healing complication) and was not able to receive dental implants. Another patient suffered from non-infectious late-term titanium mesh exposure (Class II healing complication); however, the complication was resolved, and the patient received dental implants. Seventeen out of the nineteen included patients had no surgical and healing complications [21].

#### 3.4.2. Vertical Bone Gain and Regeneration Rate

Table 5 showcases the vertical bone gains along with the regeneration rates after bone augmentation procedures, reported in the included studies. The vertical bone gain in alveolar defects was reported in all four studies 6 and/or 9 months after the alveolar ridge augmentation procedure; however, only one of the studies had reported the vertical bone gain measurements from all sides of the defects (i.e., mesial, distal, buccal, and lingual) [22,23,25,26]. The same study also reported the size and extent of the alveolar defects in terms of means and standard deviations to compare the size of the regenerated bone tissues with the initial defect sizes [22,23,25,26]. All three of the included RCTs reported a statistically insignificant difference between their study groups regarding vertical bone gain. The mean vertical bone gains ranged from 4.1 ± 1.0 mm to 6.36 ± 2.31 mm in the three included RCTs across all study groups.

All three of the RCTs reported the regeneration rates of the alveolar defects by comparing the size of the regenerated bone tissue to the initial defect size, all measured in three-dimensional CBCT imaging and reported in percentages. All three of the RCTs reported a statistically insignificant difference between their study groups regarding alveolar bone regeneration rates. The regeneration rates ranged from 39.7 ± 11.4% to 82.30 ± 17.98% in the three included RCTs across all study groups.

In the included case series, all patients received titanium meshes covered with cross-linked collagen membranes, with a mean of 8.6 ± 4.0 mm in vertical bone gain and regeneration rates of 85.8 ± 25.6% [21].

#### 3.4.3. Bone Density and Quality

Bone density was reported in only two of the studies; both studies measured the density/hardness of the newly formed bony tissues by a calibrated probing force of 30 g inserted horizontally from the buccal side of the defect [24,27,28,29]. Both studies reported statistically insignificant differences between their two study groups, with most of the cases showing a medium hardness/density (37/60, 61.66%), followed by hard (18/60, 30%) and soft (5/60, 8.33%) hardness/densities. Histological and micro-CT investigations were reported in two of the studies; both studies reported statistically insignificant differences between their study groups in regard to the amount of newly formed bone, highly mineralized bony tissues, low-mineralized bony tissues, non-mineralized tissues, and residual particles of grafting materials [22,23,24,25,26,27,28].

In the included case series, only three of the cases were selected for immunohistochemical evaluations, and all three cases demonstrated desirable ratios of newly formed bone tissue and residual grafting materials; however, no quantitative analysis was reported [21].

#### 3.4.4. Dental Implant Osseointegration and Stability

Two of the studies did not report any data on the survival rates or the osseointegration health of the dental implants placed in their patients [21,29]. Table 6 details the survival rates, osseointegration health (measured by applying a counter torque of 25 N/cm), and resonance frequency analysis (measured in implant stability quotient (ISQ)). One of the included studies reported a 100% implant survival rate for all of their cases; however, it is worth noting that this study had lost four of their patients with a total of 10 dental implants from their study due to major surgical and healing complications [22,23,25,26]. One study had reported 94.11% and 97.29% survival rates for the titanium mesh alone and the titanium mesh with cross-linked collagen membranes groups, respectively [24,27,28]. Osseointegration was only investigated in two of the studies, and both studies indicated thresholds of 35 N/cm and 15 N/cm of counter torque resistance to classify the osseointegration level: >35 N/cm (hard); 15 to 35 N/cm (medium); and <15 N/cm (low).

### 3.5. Quality Assessments

Two of the RCTs had an overall low risk of bias, while one of them had some concerns regarding missing outcome data (Supplementary Figure S1, Supplementary Table S2). The included case series had an overall moderate risk of bias; there were some concerns regarding the selection of the participants (Supplementary Figure S2, Supplementary Table S3).

## 4. Discussion

This systematic review was designed to gather all human studies that reported outcome differences in various titanium meshes and titanium-reinforced perforated membranes (e.g., dPTFE), with and without cross-linked or non-cross-linked collagen membranes. As mentioned in the introduction of this paper, clinicians often fear that if they do not apply a layer of collagen membrane on top of their titanium mesh or perforated titanium-reinforced dPTFE membranes, their regenerative treatment will lack the cell-selective properties of collagen membranes; however, the findings of the current systematic review suggest otherwise. All of the study groups of the three included RCTs showed significant mean vertical bone gains (>4 mm), which aligns with the literature standards in GBR-based VRA [3].

In a systematic review and network meta-analysis published in 2022 by Zhang et al., it was concluded that non-perforated titanium-reinforced dPTFE membranes had the highest overall vertical bone gain capabilities compared to other meshes/membranes and their combinations [10]. Zhang et al. also reported that there were no significant differences regarding both the vertical bone gain and incidence rates of surgical and healing complications between titanium mesh alone and titanium mesh + non-cross-linked collagen membranes. Zhang et al. have included both randomized clinical trials and case series in their analyses, with their included studies having various study designs/goals (i.e., different studies had investigated different kinds and combinations of meshes and membranes with methodological heterogeneities), surgical procedures, bone graft materials, and evaluation methods and periods. In addition, two of the included RCTs (seven papers) in the current systematic review were published after Zhang et al.’s systematic review, which further justified the execution of the current systematic review.

Reports of the included RCTs in the current systematic review showed no significant differences regarding vertical and horizontal bone gain, bone regeneration rates, bone quality/hardness, surgical and healing complications, dental implant survival rates, and dental implant stability and osseointegration between their study groups. The only significant difference reported across all included studies was related to the thickness and quality of pseudo-periosteum, which was significantly thinner and more vascularized (i.e., Type 1 pseudo-periosteum) in the study group that had their titanium-reinforced perforated dPTFE membranes covered with non-cross-linked collagen membranes [29]. In regard to the included case series, all patients had received titanium meshes with cross-linked collagen membranes, and overall, desirable outcomes of vertical bone gain and low rates of surgical and healing complications were reported. However, since there were no comparison groups in this case series, it cannot be determined whether similar results would have occurred without the collagen membranes or not [21].

Previous non-randomized studies have reported that GBR-based VRA procedures using CAD/CAM customized titanium meshes with accurate fitting, in situ adaptation, and no collagen membranes on top, might suffer from early or late exposures and inadequate regenerated bone volume, potentially caused by the formation of a thick layer of pseudo-periosteum connective tissue [31,32]. On the contrary, in an RCT by Cucchi et al., it was shown that there are no significant differences in vertical bone gain and surgical/healing complication incidence rates between patients who received CAD/CAM customized titanium meshes with or without a layer of cross-linked collagen membrane on top [28]. In a randomized in vivo study conducted on rabbit models, it was reported that regenerated bone volumes were significantly higher when no collagen membranes were used on top of the titanium meshes; even though the collagen membranes helped reduce the pseudo-periosteum thickness, they had no positive effects on vertical bone gain [33]. In another animal study executed in 2021 by Paeng et al., five mandibular alveolar ridge defects were created in six canine models, and the defects were either left empty (i.e., control negative) or filled and covered with different combinations of bone substitutes, titanium mesh, and collagen membrane. It was reported that defects that were covered with titanium mesh alone had the highest volumes of vertical bone gain [34]. These animal studies support the reported outcomes of the current systematic review that GBR-based VRA, using titanium mesh alone or perforated titanium-reinforced dPTFE membranes alone, results in greater vertical bone gains than when covered with collagen membranes.

In an RCT by Urban et al., the regenerative capabilities of the newly introduced titanium-reinforced perforated dPTFE membranes were investigated with and without a layer of collagen membrane on top [29]. Their results showed no significant differences between the two groups regarding vertical bone gain and incidence rates of surgical and healing complications. The incidence rates of surgical and healing complications in both groups were less than 7%, which complies with the previous findings of the systematic review and meta-analysis conducted by the same authors on non-absorbable meshes/membranes [3]. The reported incidence rates of surgical and healing complications are considerably lower than those reported in previous studies that focused on titanium meshes [22]. This reduction in complication rates could be due to the dual functionality of these newly introduced titanium-reinforced perforated dPTFE membranes. Their titanium reinforcement and presence of numerous macropores help them act similarly to titanium meshes and facilitate constructive interrelations between the flap periosteum and the treated area [35,36]. The results of this systematic review align with another meta-analysis by Gu et al., which reported that the type of titanium meshes and their combination with absorbable membranes do not make any significant differences in regard to titanium mesh exposure rates [37].

### 4.1. Study Limitations and Suggestions

(A) There are a number of in vivo and human clinical studies that have investigated the outcomes of various kinds of titanium meshes and titanium-reinforced membranes with or without collagen membranes on lateral bone regeneration (including buccal dehiscence defects). However, the number of human RCTs that have compared the outcomes of adding a collagen membrane to these meshes/membranes with a focus on vertical alveolar ridge augmentation is very small. The initial protocol for this systematic review was to only include human RCTs; however, due to the extremely limited number of human RCTs in the literature, the protocol was revised in order to include all randomized and non-randomized human clinical studies (excluding case reports). At the end, only four studies met the inclusion criteria: three RCTs and one case series. Hence, this systematic review was faced with all kinds of heterogeneities in the methodologies of these studies: defect locations (i.e., maxilla or mandible, anterior or posterior, free-end zone or supported by other teeth on both sides); defect sizes (i.e., mesio-distal extent, bucco-lingual width, and maximum vertical bone loss in the mesial, distal, buccal, and lingual sides of the defect); grafting materials (e.g., autograft + xenograft, autograft + allograft, xenograft alone, etc.); meshes and membranes (i.e., custom CAD-CAM titanium meshes, prefabricated titanium meshes, perforated titanium-reinforced dPTFE, non-perforated titanium-reinforced dPTFE, cross-linked collagen, and non-cross-linked collagen membranes); surgical protocol (i.e., dental implantation months after GBR (two-staged), or simultaneous dental implantation with GBR (one-staged)). Given the current status of the literature with a limited number of RCTs on this topic and with heterogeneities in many methodological aspects of the studies, it is fair to say that the literature requires further similar studies to reach a reliable conclusion on the superiorities/inferiorities of these surgical procedures and regenerative materials.

(B) In most cases, the measurements of the vertical bone gain were reported in an overall mean of the most vertically resorbed area of the alveolar bone rather than a detailed reporting of each side of the defect (i.e., mesial, distal, buccal, and lingual sides). Moreover, the vertical and horizontal bone gain measurements were also mostly reported in overall means and were not specified for each side of the defect. Researchers and clinicians are highly encouraged to precisely report both the defect sizes and vertical/horizontal bone gains in all sides of the defect, as well as the most vertically resorbed point.

(C) One of the key factors that needs to be evaluated after GBR-based VRA is the quality and thickness of the pseudo-periosteum formed beneath the mesh/membrane, specifically in cases of titanium meshes or perforated membranes. Unfortunately, only two of the included studies reported the type of pseudo-periosteum measured while removing the non-resorbable meshes/membranes. Future researchers are highly encouraged to follow the pseudo-periosteum thickness reporting protocols in future clinical studies.

(D) Out of the four included studies, only two reported both the absolute and relative vertical bone gain measurements [21,29], whilst the other two reported only the absolute vertical bone gain measurements. It is crucial for researchers and clinicians to keep in mind that the initial size and dimensions of the vertical alveolar ridge defect will directly determine the full potential of the relative vertical bone gain in their regenerative treatment of the defect. In most cases, the bone peak adjacent to the alveolar defect will dictate/determine the maximum regenerative potential of the treatment. In a case series executed by Urban et al. in 2021, it was concluded that vertical bone losses < 5 mm exhibit much greater relative vertical bone gains compared to critically sized defects (i.e., >8 mm) when using titanium-reinforced dPTFE membranes covered with native non-cross-linked collagen membranes; even though the absolute vertical bone gain is usually bigger in the critically sized defects [38]. Researchers and clinicians are highly encouraged to keep this factor in mind and try to report both the relative and absolute vertical bone gains in future similar studies.

(E) Due to the listed reasons, conducting a meta-analysis was not feasible for this systematic review. Adequate randomized human clinical trials in the literature need to have relatively similar methodologies in order to conduct a proper meta-analysis and hopefully reach a reliable conclusion regarding the best combination of meshes and membranes for GBR-based VRA.

(F) Using either titanium mesh alone or perforated titanium-reinforced dPTFE membranes alone in GBR-based VRA leads to significant vertical bone gain volumes. In order to reach firm and reliable conclusions on this debatable topic, it is highly suggested that future human clinical studies, designed and executed in a randomized setting, have four study groups: (1) titanium mesh alone; (2) titanium mesh with cross-linked collagen membrane; (3) perforated titanium-reinforced dPTFE membrane alone; and (4) perforated titanium-reinforced dPTFE membrane with cross-linked collagen membrane.

(G) One of the main reasons that some clinicians prefer to apply collagen membranes is to reduce the chances of flap dehiscence. The three included RCTs reported no meaningful differences in surgical or healing complication rates between their study groups (with and without collagen membranes). This might be due to the fact that these reports were only from experts in the field who might not necessarily need collagen membranes to help them prevent flap dehiscence. However, reports regarding general practitioners are not available in the literature.

### 4.2. Conclusions

The addition of absorbable collagen membranes on top of titanium meshes or perforated titanium-reinforced dPTFE membranes does not lead to higher vertical bone gains or less surgical and healing complication rates in GBR-based VRA. Collagen membranes might be able to limit the thickness of the pseudo-periosteum connective tissue. There are no significant differences between cross-linked and non-cross-linked collagen membranes regarding bone regeneration capabilities or incidence of complications. The newly introduced perforated titanium-reinforced dPTFE membranes carry the same regenerative capabilities of titanium meshes while also benefiting from desirable secondary closure capabilities. There is still a void in the literature for more human RCTs with broader study groups to determine the superiorities/inferiorities of titanium meshes and perforated titanium-reinforced dPTFE membranes.

## Figures and Tables

**Table 1:** Search queries.

Data Base	Date	Search Query	Results
Medline via PubMed	5 June 2025	(“Alveolar Ridge Augmentation” [MeSH] OR “Ridge Augmentation” OR “Bone Regeneration” [MeSH] OR “Bone Regeneration”) AND (“Collagen Membrane” OR (“Collagen” AND “Membrane”) OR “Resorbable Membrane” OR (“Resorbable” AND “Membrane”)) AND (“Surgical Mesh” [MeSH] OR “Mesh” OR “Titanium Mesh” OR (“Titanium” AND “Mesh”) OR “Ti-Reinforced Mesh” OR (“Ti-Reinforced” AND “Mesh”))	65
Scopus	5 June 2025	TITLE-ABS-KEY (bone AND augmentation OR alveolar AND ridge AND augmentation) AND (collagen) AND (titanium AND mesh OR titanium OR mesh OR membrane)	1039
Web of Science	5 June 2025	(ALL = (alveolar ridge augmentation) OR ALL = (bone regeneration) OR ALL = (ridge augmentation) OR ALL = (guided bone regeneration)) AND (ALL = (collagen membrane) OR ALL = (resorbable membrane)) AND (ALL = (titanium mesh) OR ALL = (Ti-reinforced mesh) OR ALL = (Ti-reinforced membrane) OR ALL = (mesh))	183
Embase	5 June 2025	(Alveolar ridge) AND (Collagen) AND (Titanium OR Mesh OR Membrane)	347
Cochrane Library	5 June 2025	Alveolar Ridge Augmentation [MeSH] AND Surgical Mesh [MeSH] AND Collagen	15

**Table 2:** Treatment plans and in-study comparisons.

Groups	Meshes and Membranes	Patients	Studies
1	Titanium mesh	15	1 [24,27,28]
2	Titanium mesh + cross-linked collagen	35	2 [22,23,24,25,26,27,28]
3	Non-perforated Ti-reinforced dPTFE	20	1 [22,23,25,26]
4	Perforated Ti-reinforced dPTFE	15	1 [29]
5	Perforated Ti-reinforced dPTFE + non-cross-linked collagen	15	1 [29]
6	Titanium mesh + cross-linked collagen (***Case Series***)	19	1 [21]

***Abbreviations:*** Titanium (Ti) and dense polytetrafluoroethylene (dPTFE). ***Note:*** The comparisons made in the 3 included RCTs were between the following study groups: 1 vs. 2, 2 vs. 3, and 4 vs. 5.

**Table 3:** Study variables, evaluation methods, and periods.

Number	Study Variable	Evaluation Method	Evaluation Period	References
1	Surgical and healing complications	The Fontana et al.’s classification	T6	1 [24,27,28]
			T9	2 [22,23,25,26,29]
2	Pseudo-periosteum	Clinical examination	T6	1 [24,27,28]
			T9	1 [29]
3	Vertical bone gain	CBCT	T6	1 [24,27,28]
			T9	2 [22,23,25,26,29]
4	Regeneration rate	CBCT	T6	1 [24,27,28]
			T9	1 [29]
5	Bone quality	Histology and micro CT (sampling at least 4 mm of depth with a trephine bur)	T9	2 [22,23,24,25,26,27,28]
6	Implant osseointegration	Reverse (counter) torque at 25 N/cm	T9: 9 months after bone augmentation and 3 months after implantation	1 [24,27,28]
			T9: 9 months after bone augmentation and implantation	1 [22,23,25,26]
7	Implant stability and survival	Resonance frequency analysis in ISQ	T9: 9 months after bone augmentation and 3 months after implantation	1 [24,27,28]
			T9: 9 months after bone augmentation and implantation	1 [22,23,25,26]

***Abbreviations*:** Cone beam computed tomography (CBCT) and implant stability quotient (ISQ).

**Table 4:** Surgical and healing complications and pseudo-periosteum.

Groups	Meshes and Membranes	Surgical Complications ‡			Healing Complications ‡				Pseudo-Periosteum §		
		Class A	Class B	Class C	Class I	Class II	Class III	Class IV	Type 1	Type 2	Type 3
1	Titanium mesh	1/15	1/15	-	-	2/15	1/15	2/15	7/15	4/15	4/15
2	Titanium mesh + cross-linked collagen	2/34	6/34	-	-	2/34	3/34	1/34	10/15 **†**	4/15 **†**	1/15 **†**
3	Non-perforated Ti-reinforced dPTFE	-	1/20	-	-	1/20	1/20	1/20	NS	NS	NS
4	Perforated Ti-reinforced dPTFE	-	-	-	1/15	-	-	-	4/15 *	10/15	1/15
5	Perforated Ti-reinforced dPTFE + non-cross-linked collagen	-	-	-	1/15	-	-	-	11/15 *	4/15	0/15
6	Titanium mesh + cross-linked collagen (***Case Series***)	-	-	-	-	1/19	-	1/19	NS	NS	NS

***Abbreviations:*** Not specified (NS); titanium (Ti); and dense polytetrafluoroethylene (dPTFE). ***Note:*** The comparisons made in the 3 included RCTs were between the following study groups: 1 vs. 2, 2 vs. 3, and 4 vs. 5. **‡:** Both the surgical and healing complications were evaluated and examined based on Fontana et al.’s (2011) [8] classifications for surgical and healing complications in alveolar ridge augmentations. **§:** The thickness and quality of the pseudo-periosteum connective tissue were evaluated according to Cucchi et al.’s (2019) [30] classification. **†:** The combination of Titanium mesh with cross-linked collagen membrane was used in three of the included studies; however, pseudo-periosteum thickness was only investigated and reported in one of the studies with 30 cases total, with 15 of them being in the Titanium mesh with cross-linked collagen membrane group. * The difference between these two groups was statistically significant.

**Table 5:** Vertical bone gain and regeneration rates.

Groups	Meshes and Membranes	Vertical Bone Gain (Mean ± SD (mm)) *					Regeneration Rate	References
		Mesial	Distal	Buccal	Lingual	Overall		
1	Titanium mesh	NS	NS	NS	NS	4.74 ± 2.56	74.32 ± 22.10%	[24,27,28]
2	Titanium mesh + cross-linked collagen	NS	NS	NS	NS	6.36 ± 2.31	82.30 ± 17.98%	
		3.3 ± 1.0	4.0 ± 1.0	5.1 ± 1.4	3.8 ± 0.8	4.1 ± 1.0	42.1 ± 18.1% **†**	[22,23,25,26]
3	Non-perforated Ti-reinforced dPTFE	3.6 ± 1.2	4.1 ± 1.0	5.0 ± 1.0	4.2 ± 1.2	4.20 ± 1.00	39.7 ± 11.4% **†**	[22,23,25,26]
4	Perforated Ti-reinforced dPTFE	NS	NS	NS	NS	4.47 ± 2.05	69.30 ± 17.90%	[29]
5	Perforated Ti-reinforced dPTFE + non-cross-linked collagen	NS	NS	NS	NS	4.11 ± 2.69	72.3 ± 16.4%	
6	Titanium mesh + cross-linked collagen (***Case Series***)	NS	NS	NS	NS	8.6 ± 4.0	85.8 ± 25.6%	[21]

***Abbreviations:*** Not specified (NS); titanium (Ti); and dense polytetrafluoroethylene (dPTFE). ***Note:*** The comparisons made in the 3 included RCTs were between the following study groups: 1 vs. 2, 2 vs. 3, and 4 vs. 5. *: The measurements for vertical bone gain were reported as mean ± SD. **†:** This study only measured the newly formed coronal bony tissues 9 months after simultaneous bone augmentation and dental implantation.

**Table 6:** Dental implant evaluations.

Groups	Meshes and Membranes	Survival Rates	Osseointegration *	Resonance Frequency Analysis **	References
1	Titanium mesh	94.11%	>35 N/cm: 66.66% <35 N/cm: 33.33%	NS	[24,27,28] **‡**
2	Titanium mesh + cross-linked collagen	97.29%	>35 N/cm: 60% <35 N/cm: 40%	NS	
		100% **†**	>35 N/cm: 100%	66.5 ± 10.00 ISQ	[22,23,25,26] **†**
3	Non-perforated Ti-reinforced dPTFE	100% **†**	>35 N/cm: 100%	71.00 ± 8.00 ISQ	[22,23,25,26] **†**

***Abbreviations:*** Not specified (NS); titanium (Ti); dense polytetrafluoroethylene (dPTFE); and implant stability quotient (ISQ). ***Note:*** The comparisons made in the 3 included RCTs were between the following study groups: 1 vs. 2, and 2 vs. 3. *: The success of osseointegration was measured by applying a counter torque of 25 N/cm. **: Resonance frequency analysis was measured in the implant stability quotient (ISQ). **‡:** In this study, the dental implants were inserted 6 months after the bone augmentation procedure and evaluated 3 months after insertion. **†:** In this study, the dental implants were placed simultaneously with the augmentation procedure and evaluated 9 months after insertion; 4 patients with a total of 10 implants were excluded from the study before implant analyses due to major surgical and healing complications.

## Data Availability

No new data were created or analyzed in this study. Data sharing is not applicable to this article.

## Supplementary Materials

The following supporting information can be downloaded at: https://www.mdpi.com/article/10.3390/jfb16070246/s1, Supplementary Table S1: Extracted data from included studies; Supplementary Table S2: Detailed quality assessment of the included randomized studies; Supplementary Table S3: Detailed quality assessment of the included non-randomized studies; Supplementary Figure S1: Risk of bias assessment for randomized studies; Supplementary Figure S2: Risk of bias assessment for non-randomized studies.
