# Supplementary material for: Accompanying Titanium Meshes and Titanium-Reinforced Membranes with Collagen Membranes in Vertical Alveolar Ridge Augmentations: A Systematic Review
Source: J Funct Biomater. 2025 Jul 4;16(7):246. doi: 10.3390/jfb16070246 (PMC12295274; doi:10.3390/jfb16070246)
Supplement: Supplementary file 1 [file jfb-16-00246-s001.zip › jfb-3662384-supplementary.pdf]

Supplementary Figure S1.

Study / *Domain*	*D1*	*D2*	*D3*	*D4*	*D5*	*Overall*
Cucchi et al. (2021 and 2024)						
Cucci et al. (2017, 2019, 2021 and 2023)						
Urban et al. (2025)						

Domains:

D1: Randomization process.
D2: Deviation from intended interventions.
D3: Missing outcome data.
D4: Measurement of the outcome.
D5: Selection of the reported result.

Judgement:

Low Risk of Bias	
Some Concerns	
High Risk of Bias	

Supplementary Figure S2.

Study / *Domain*	*D1*	*D2*	*D3*	*D4*	*D5*	*D6*	*D7*	*Overall*
Funato et al. (2013)								

Domains:

D1: Bias due to confounding.
D2: Bias in classification of interventions.
D3: Bias in selection of participants.
D4: Bias due to deviations in intended interventions.
D5: Bias due to missing data.
D6: Bias in measurement of outcomes.
D7: Bias in selection of the reported results.

Judgement:

Low Risk of Bias	
Moderate Risk of Bias	
Serious Risk of Bias	
Critical Risk of Bias	

Supplementary Table S1. Extracted data.

Author (Year) / Study Type<br><br>***A) Number of Patients***<br>***B) Gender (M:F)***<br>***C) Age***	***A) Surgical Procedure***<br>***B) Graft Material***	***A) Study Phases***<br>***B) Study Groups***<br>***C) Dental Implants***<br>***D) Alveolar Defects***	***Study Variables / Evaluation Methods (Periods)***	***Outcomes and Complications***
Cucchi et al. (2021, 2024, and 2024) (22,25,26) / Human RCT<br><br>A) 30<br><br>B) 15:15<br><br>C) > 18<br><br>***Note:*** This is a three-part study with its results spread into three papers.	A) Two-staged three-dimensional vertical and/or horizontal ridge augmentation<br><br>- Decortication<br><br>B) 0.5 – 1.0 g of autologous bone was harvested from posterior mandible<br><br>- The Titanium mesh was filled with a 50:50 mix of autogenous bone and xenograft (Zcore®, Osteogenics Biomedical)<br><br>- The Titanium meshes were fixed using osteosynthesis screws (Profix® System, Biomedical)	A)<br>A.1. The initial surgery; placement of Titanium mesh (**T0**)<br><br>A.2. Six months after the initial surgery; removal of mesh and placement of Titanium dental implants (BT Safe®, BTK, Biotec Srl, Dueville) (**T6**)<br><br>A.3. Three months after the dental implant placement (**T9**)<br><br>B)<br>B.1. Customized CAD/CAM Titanium mesh (n = 15) (Group 1)<br><br>B.2. Customized CAD/CAM Titanium mesh with cross-linked collagen membranes (Cytoplast® RTM, Osteogenics Biomedical) (n = 15) (Group 2)<br><br>C) 71 implants; 34 and 37 implants for Group 1 and Group 2, respectively.<br><br>D)<br>D.1. Group 1:<br>- Maxilla (n = 5)<br>- Mandible (n = 10)<br>- Anterior (n = 2)<br>- Posterior (n = 13)<br><br>D.2. Group 2:<br>- Maxilla (n = 9)<br>- Mandible (n = 6)<br>- Anterior (n = 4)<br>- Posterior (n = 11)<br><br>D.3. All patients:<br>- Vertical defects (n = 25)<br>- Horizontal defects (n = 5)	1. Surgical and healing complications / Clinical evaluation (Fontana et al.'s classification) (T0, T6 and T9)<br><br>2. Exposure and infection of the mesh / Clinical evaluation (T6)<br><br>3. Pseudo-periosteum thickness / UNC-15 probe (T6)<br><br>4. Bone density / Calibrated probing force of 30 gm (T6)<br><br>5. Bone volume / CBCT (T0 and T6)<br><br>6. Regeneration rate / CBCT (T6)<br><br>7. Vertical bone gain / CBCT (T6)<br><br>8. Implant survival and osseointegration / Counter torque of 25 N/cm (T9)<br><br>9. Bone quality / Histological, histomorphometric, and Micro-CT; using a 4-mm-diameter trephine bur, reaching a depth of at least 4 mm (T6)	1. Surgical/technical complications:<br>No significant differences between the two groups (P = 0.65):<br>- Group 1: n = 2, 13.3%<br>- Group 2: n = 4, 26.7%<br>- Overall, there were no failures flap advancement and primary closure. Four paresthesia lesions occurred bet were all treated. One partial mesh fracturing and one partial mesh misfitting occurred.<br><br>2. Healing complications:<br>No significant differences between the two groups (P = 0.39):<br>- Group 1: n = 5 (33.3%)<br>- Group 2: n = 2 (13.3%)<br>- Three early and two late exposures of the meshes (two class 2 and three class 3) and two infections without exposure (two class 4) occurred. All cases were managed before dental implantation.<br><br>3. Pseudo-periosteum:<br>No significant differences between the two groups (P = 0.46):<br>3.1. Type 1:<br>- Group 1: n = 7 (46.7%)<br>- Group 2: n = 10 (66.7%)<br>3.2. Type 2:<br>- Group 1: n = 4 (26.7%)<br>- Group 2: n = 4 (26.7%)<br>3.3. Type 3:<br>- Group 1: n = 4 (26.7%)<br>- Group 2: n = 1 (6.7%)<br><br>4. Bone density:<br>No significant differences between the two groups (P = 0.75):<br>4.1. Hard:<br>- Group 1: n = 4 (26.7%)<br>- Group 2: n = 3 (20%)<br>4.2. Medium:<br>- Group 1: n = 10 (66.7%)<br>- Group 2: n = 9 (60%)<br>4.3. Soft:<br>- Group 1: n = 1 (6.7%)<br>- Group 2: n = 3 (20%)

Author (Year) / Study Type<br><br>*A) Number of Patients*<br>*B) Gender (M:F)*<br>*C) Age*	*A) Surgical Procedure*<br>*B) Graft Material*	*A) Study Phases*<br>*B) Study Groups*<br>*C) Dental Implants*<br>*D) Alveolar Defects*	*Study Variables / Evaluation Methods (Periods)*	*Outcomes and Complications*
				5. Implant stability:<br>No significant differences between the two groups (P = 1.00):<br>5.1. > 35 N:<br>- Group 1: n = 10 (66.7%)<br>- Group 2: n = 9 (60%)<br>5.2. < 35 N:<br>- Group 1: n = 5 (33.33%)<br>- Group 2: n = 6 (40%)<br>5.3. Out of the 71 implants, 43 implants had high stability (> 35 N), 27 had medium stability (35 – 15 N), and only one implant had a torque value less than 15 N.<br><br>6. Regenerated bone volume:<br>No significant differences between the two groups (P = 0.53):<br>6.1. Group 1:<br>- Mean ± SD: 803.07 mm^3 ± 553.78 mm^3<br>- Median: 759 mm^3, 95% CI: 496.40 mm^3; 1109.74 mm^3<br>6.2. Group 2:<br>- Mean ± SD: 843.13 mm^3 ± 389.52 mm^3<br>- Median: 766 mm^3, 95% CI: 627.42 mm^3 - 1058.8 mm^3<br><br>7. Regeneration rates:<br>No significant differences between the two groups (P = 0.44):<br>7.1. Group 1:<br>- Mean ± SD: 74.32% ± 22.10%<br>- Median: 74.54%, 95% CI: 62.08% - 86.55%<br>7.2. Group 2:<br>- Mean ± SD: 82.30% ± 17.98%<br>- Median: 88.05%, 95% CI: 72.34% - 92.26%<br><br>8. Vertical bone gain:<br>No significant differences between the two groups (P = 0.11):<br>8.1. Group 1:<br>- Mean ± SD: 4.74 mm ± 2.56 mm<br>- Median: 4.02 mm, 95% CI: 3.02 mm – 6.46 mm<br>8.2. Group 2:<br>- Mean ± SD: 6.36 mm ± 2.31 mm<br>- Median: 6.28 mm, 95% CI: 5.03 mm – 7.69 mm<br><br>9. Implant survival rates:<br>Three implants failed to achieve osseointegration; two implants in one patient in Group 1, and one implant in Group 2. No significant differences between the two groups (P = 1.00):

Author (Year) / Study Type <br>***A) Number of Patients*** ***B) Gender (M:F)*** ***C) Age***	***A) Surgical Procedure*** ***B) Graft Material***	***A) Study Phases*** ***B) Study Groups*** ***C) Dental Implants*** ***D) Alveolar Defects***	***Study Variables / Evaluation Methods (Periods)***	***Outcomes and Complications***
				9.2. Group 1: 94.0% 9.3. Group 2: 97.0% <br>10. Two cases needed GBR before implant placement. In all the other cases, implants were placed as planned. <br>11. Histological evaluation: Out of the 30 cases, the biopsies of only 20 cases were evaluated; Group 1 (n = 9) and Group 2 (n = 11). The biopsies of 5 cases were not collected due to their healing complications, and 5 other cases could not be investigated due to the very limited quantity of their biopsies. No significant differences between the two groups in the amount of newly formed bone tissue and non-mineralized tissue. Bone regeneration was observed in all samples. <br>12. Micro-CT analysis: Five patients were excluded due to healing complications; 2 in Group 1 and 3 in Group 2. No significant differences between the two groups regarding high-mineralized bone, low-mineralized bone, residual particles of grafting material, and non-mineralized tissue volumes. <br>**<u>Conclusions:</u>** Both methods are reliable solutions for vertical and horizontal augmentation before implantation. The addition of collagen membranes seems to favor a better trend in regeneration and healing complication rates, however, there were no statistically significant difference between the two groups regarding any of the analyses.
Cucchi et al. (2017, 2019, 2021, and 2023) (20,21,23,24) / Human RCT <br>A) 40 <br>B) 13:27 <br>C) Mean age: 52	A) One-staged three-dimensional augmentation of vertical and/or horizontal defects and dental implantation <br>- Decortication <br>B) 0.5–1.0 g of autogenous bone was harvested from the external oblique ridge of the mandibular ramus using a bone scraper (Safescraper, Meta, RE, Italy); 50:50 mix of autogenous bone	A) A.1. Simultaneous bone augmentation and dental implantation (**T0**) <br>A.2. Removal of the Titanium mesh and membranes 9 months after the surgery (**T9**) <br>A.3. Functional implant loading and prosthesis placement (**T11-T12**) <br>A.4. Long-term evaluation of augmented sites and their implants at 1 year (**T24**) and 3 years (**T48**) post-prosthesis	1. Surgical and healing complications / Clinical examination (Fontana et al.'s classification) (T9) <br>2. Vertical bone gain / Measured at 4 different sites (mesial, distal, buccal, and lingual) by UNC-15 probes and calculating the difference between these measurements at T0 and T9 (T0 and T9) <br>3. Implant stability / Insertion torque expressed in N/cm and resonance frequency analysis (RFA) in ISQ (T0 and T9) <br>4. Osseointegration / Reverse	1. One patient with 3 implants was dropped out of study due to maxillofacial traumatic accident occurring before the T9 follow up. <br>2. Three patients with 7 total implants were dropped out of the study due to major complications and needed the removal of barriers, meshes and implants before T9. <br>3. In total, the data of 39 patients were recorded from T0 to T9; 20 in Group 1 and 19 in Group 2. Finally, the data of 36 patients were reported at T9; 19 in Group 1 and 17 in Group 2. <br>4. Implant stability and osseointegration: 4.1. T0:

Author (Year) / Study Type<br><br>***A) Number of Patients***<br>***B) Gender (M:F)***<br>***C) Age***	***A) Surgical Procedure***<br>***B) Graft Material***	***A) Study Phases***<br>***B) Study Groups***<br>***C) Dental Implants***<br>***D) Alveolar Defects***	***Study Variables / Evaluation Methods (Periods)***	***Outcomes and Complications***
**<u>* Note:</u>** This is a four-part study with its results spread into four papers.	and allograft (EnCore, Osteogenics Biomedical, Lubbock, Texas)	B)<br>B.1. Non-perforated titanium-reinforced d-PTFE membranes (Cytoplast Ti-250XL; Osteogenics Biomedical) (n = 20) (Group 1)<br><br>B.2. Titanium meshes (Trinon Titanium; Karlsruhe, Germany) covered by cross-linked collagen membranes (Osseoguard, Zimmer Biomet, Warsaw, Indiana) (n = 20) (Group 2)<br><br>C) 108 implants (BT SAFE; Biotec srl, Vicenza, Italy); all placed in Type I and II bone (Misch classification).<br><br>D) Posterior mandible (n = 40); no significant differences between the two groups regarding the means of the defect sizes (P = 0.29)<br>D.1. Group 1 means:<br>- Mesial: 3.2 ± 0.9<br>- Distal: 3.5 ± 0.8<br>- Buccal: 4.8 ± 0.7<br>- Lingual: 3.6 ± 0.9<br>- Mean: 3.8 ± 0.7<br>D.2. Group 2 means:<br>- Mesial: 3.1 ± 0.9<br>- Distal: 3.8 ± 1.0<br>- Buccal: 5.7 ± 1.6<br>- Lingual: 3.4 ± 0.8<br>- Mean: 4.0 ± 0.8	torque at 25 N/cm (T9)<br><br>5. Bone quality / Histological, histomorphometric, and Micro-CT; using a 4-mm-diameter trephine bur, reaching a depth of at least 4 mm (T9)	4.1.1. Out of the 108 implants, 106 (97.2%) showed optimal primary stability; mean insertion torque at 80 ± 10 N/cm and mean RFA at 86.5 ± 5.0 ISQ.<br>4.1.2. Group 1: Mean insertion torque at 80.0 ± 10.0 N/cm and mean RFA at 87.0 ± 5.0 ISQ.<br>4.1.3. Group 2: Mean insertion torque at 79.0 ± 10.0 N/cm and mean RFA at 84.5 ± 6.0 ISQ.<br>4.1.4. No significant differences between the 2 groups at T0.<br>4.2. T9:<br>4.2.1. Group 1: Mean RFA at 71.0 ± 8.0 ISQ<br>4.2.2. Group 2: Mean RFA at 66.5 ± 10.0 ISQ<br><br>5. Surgical complications:<br>5.1. Class B (i.e., neurological) complications happened in 4 cases at T0; 1/20 in Group 1 and 3/19 in Group 2. All cases showed spontaneous recovery during the first month post-surgery.<br>5.2. No complications occurred at T9<br>5.3. No significant differences between the two groups (P = 0.34)<br><br>6. Healing complications:<br>6.1. In total, 7 complications occurred from T0 to T9; 3/20 in Group 1 and 4/19 in Group 2.<br>6.2. Group 1: Two of these complications were either Class III or IV and affected the success of bone augmentation and were considered major complications. One complication was Class II and was considered minor.<br>6.3. Group 2: Three of these complications were either Class III or IV and affected the success of bone augmentation and were considered major complications. One complication was Class II and was considered minor.<br>6.4. No significant differences between the two groups (P = 0.69)<br><br>7. Vertical bone gain:<br>7.1. No significant differences between the two groups regarding the means of the dimensions of vertical bone gain (P = 0.58)<br>7.2. Group 1 means:<br>- Mesial: 3.6 ± 1.2 mm<br>- Distal: 4.1 ± 1.0 mm<br>- Buccal: 5.0 ± 1.0 mm<br>- Lingual: 4.2 ± 1.2 mm<br>- Mean: 4.2 ± 1.0 mm<br>7.3. Group 2 means:<br>- Mesial: 3.3 ± 1.0 mm

Author (Year) / Study Type<br><br>***A) Number of Patients***<br>***B) Gender (M:F)***<br>***C) Age***	***A) Surgical Procedure***<br>***B) Graft Material***	***A) Study Phases***<br>***B) Study Groups***<br>***C) Dental Implants***<br>***D) Alveolar Defects***	***Study Variables / Evaluation Methods (Periods)***	***Outcomes and Complications***
				- Distal: 4.0 ± 1.0 mm<br>- Buccal: 5.1 ± 1.4 mm<br>- Lingual: 3.8 ± 0.8 mm<br>- Mean: 4.1 ± 1.0 mm<br>7.4. Group 1: 63% (n = 34) of implants showed supra-implant bone overgrowth at T1 and an osteotomy was needed to place the healing screws. Only 25.9% (n = 14) of implants showed partial bone augmentation with the presence of residual bone defects, all in the buccal sites.<br>7.5. Group 2: 61.4% (n = 27) of implants showed supra-implant bone overgrowth at T1 and an osteotomy was needed to place the healing screws. Only 22.7% (n = 10) of implants showed partial bone augmentation with the presence of residual bone defects, all in the buccal sites.<br><br>8. Histological evaluation:<br>Four cases were excluded from these analyses due to healing complications. Six of the cases could not be sampled due to the risk of damaging the bone surrounding implants. Five cases were excluded since the amount of regenerated bone was < 1 mm. A total of 25 cases were sampled.<br>No significant differences between the two groups regarding the percentages of the coronal newly-formed bone tissue percentages (P = 0.72);<br>8.1. Group 1 (n = 13): 39.7 ± 11.4 %<br>8.2. Group 2 (n = 12): 42.1 ± 18.1 %<br><br>**Conclusions:**<br>The use of double-variable tapered implants is reliable in both immediate GBR approaches. There were no statistically significant differences between the two groups in any of the outcomes.
Urban et al. (2025) (27) / Human RCT<br><br>A) 30<br><br>B) 9:21<br><br>C) Mean age: 51.2 ± 10.6	A) Two-staged three-dimensional augmentation of vertical and/or horizontal defects<br><br>- Decortication<br><br>B) Autogenous bone was harvested from posterior mandibular using a bone scraper (Safescraper, Meta, RE, Italy); 50:50 mix of autogenous bone	A)<br>A.1. The initial surgery; placement of Titanium-reinforced mesh (**T0**)<br><br>A.2. Nine months after the initial surgery; removal of membranes and placement of Titanium dental implants (**T9**)<br><br>B)	1. Absolute and relative vertical bone gain / CBCT (T0 and T9)<br><br>2. Horizontal bone gain / CBCT (T0 and T9)<br><br>3. Regeneration rate / CBCT (T0 and T9)<br><br>4. Pseudo-periosteum / UNC-15 probe (T9)	1. Absolute vertical bone gain:<br>No significant differences between the two groups (P = 0.680);<br>- Group 1: 4.47 ± 2.05 mm<br>- Group 2: 4.11 ± 2.69 mm<br><br>2. Relative vertical bone gain:<br>No significant differences between the two groups (P = 0.436);<br>- Group 1: 79.2 ± 16.6 %<br>- Group 2: 85.8 ± 10.6 %

Author (Year) / Study Type<br><br>***A) Number of Patients***<br>***B) Gender (M:F)***<br>***C) Age***	***A) Surgical Procedure***<br>***B) Graft Material***	***A) Study Phases***<br>***B) Study Groups***<br>***C) Dental Implants***<br>***D) Alveolar Defects***	***Study Variables / Evaluation Methods (Periods)***	***Outcomes and Complications***
	and deproteinized bovine bone mineral (DBBM; Bio-Oss, Geistlich Pharma AG)	B.1. Perforated titanium- reinforced d-PTFE membrane (RPM, Osteogenics Biomedical) (n = 15) (Group 1)<br>B.2. Perforated titanium- reinforced d-PTFE membrane (RPM, Osteogenics Biomedical) with collagen membrane (Bio- Gide, Geistlich Pharma AG) (n = 15) (Group 2)<br><br>C) No details were reported regarding the kind of the titanium implants, or the number of implants used.<br><br>D)<br>D.1. Defect locations in Group 1:<br>- Posterior mandible: 9<br>- Posterior maxilla: 4<br>- Anterior mandible: 1<br>- Anterior maxilla: 1<br>D.2. Defect locations in Group 2:<br>- Posterior mandible: 6<br>- Posterior maxilla: 2<br>- Anterior mandible: 1<br>- Anterior maxilla: 6<br>D.3. Means of vertical defects:<br>- Group 1: 5.59 ± 2.11 mm<br>- Group 2: 4.75 ± 2.95 mm<br>D.4. Means of mesio-distal lengths of the defects:<br>- Group 1: 20.07 ± 7.63 mm<br>- Group 2: 20.07 ± 6.67 mm	5. Bone density / Based on the extension of probe penetration into the newly-formed bone (T9)<br><br>6. Healing complications / Clinical evaluations (Fontana et al.'s classification) (T9)	3. Horizontal bone gain:<br>No significant differences between the two groups (P = 0.338);<br>- Group 1: 10.34 ± 2.16 mm<br>- Group 2: 9.67 ± 1.50 mm<br><br>4. Augmented bone volume<br>No significant differences between the two groups (P = 0.057);<br>- Group 1: 2229.8 ± 980 mm^3<br>- Group 2: 1595.3 ± 752.8 mm^3<br><br>5. Regenerated bone volume:<br>No significant differences between the two groups (P = 0.188);<br>- Group 1: 1649.1 ± 1066.6 mm^3<br>- Group 2: 1201.3 ± 719 mm^3<br><br>6. Lacking bone volume:<br>Significantly higher in Group 1 (P = 0.048);<br>- Group 1: 580.7 ± 264.8 mm^3<br>- Group 2: 394 ± 229.4 mm^3<br><br>7. Effective regeneration rate:<br>No significant differences between the two groups (P = 0.630);<br>- Group 1: 69.3 ± 17.9 %<br>- Group 2: 72.3 ± 16.4 %<br><br>8. Healing complications:<br>No significant differences between the two groups (P = 1.000);<br>- Group 1: 1 (6.7%)<br>- Group 2: 1 (6.7%)<br>- Both cases were Class I complications (mesh exposures 6 months after surgery)<br><br>9. Pseudo-periosteum:<br>The number of cases with Type 1 pseudo-periosteum was significantly higher in Group 2 (P = 0.014)<br>9.1. Type 1:<br>- Group 1: 4 (26.7%)<br>- Group 2: 11 (73.3%)<br>9.2. Type 2:<br>- Group 1: 10 (66.7%)<br>- Group 2: 4 (26.7%)<br>9.3. Type 3:<br>- Group 1: 1 (6.7%)

Author (Year) / Study Type<br><br>***A) Number of Patients***<br>***B) Gender (M:F)***<br>***C) Age***	***A) Surgical Procedure***<br>***B) Graft Material***	***A) Study Phases***<br>***B) Study Groups***<br>***C) Dental Implants***<br>***D) Alveolar Defects***	***Study Variables / Evaluation Methods (Periods)***	***Outcomes and Complications***
				- Group 2: 0 (0%)<br><br>10. Bone density:<br>No significant differences between the two groups (P = 0.705)<br>10.1. High:<br>- Group 1: 5 (33.3%)<br>- Group 2: 6 (40.0%)<br>10.2. Medium:<br>- Group 1: 9 (60.0%)<br>- Group 2: 9 (60.0%)<br>10.3. Low:<br>- Group 1: 1 (6.7%)<br>- Group 2: 0 (0%)<br><br>11. None of the cases in both groups needed additional bone augmentation (P = 1.000). All implant sites exhibited a bone wall thickness ≥ 1.5 mm in<br>both the buccal and lingual/palatal aspects.<br><br><br>**<u>*Conclusions:</u>**<br>The addition of a collagen membrane may help prevent soft tissue ingrowth; however, it does not have any effect on vertical bone gain or surgical and healing complication rates.
Funato et al. (2013) / Human Case Series<br><br>A) 19<br><br>B) 10:9<br><br>C) Mean age: 50.2 ± 14.4, Age range: 17 – 68	A) Two-staged three-dimensional augmentation of vertical and/or horizontal defects<br><br>B) The titanium meshes with 0.1 mm thickness were pre-shaped on study casts. Bovine bone (0.25 – 1 mm particles, BioOss, OsteoHealth) and autologous bone harvested from posterior mandible were mixed in a 1:1 or 4:1 ratio. All mixed with rhPDGF-BB.	A)<br>A.1. The initial surgery; placement of titanium meshes and collagen membranes (**T0**)<br><br>A.2. Six months (or more) after the initial surgery; removal of membranes and placement of titanium dental implants (**T6 – T8**); this stage was executed when the healing period of the wounds were completed.<br><br><br>B) All cases received pre-shaped titanium meshes covered with cross-linked collagen membranes (Ossix Plus, Oraphrama).<br><br>C) Not specified.	1. Vertical bone gain / Clinical examination using UNC-15 probe and comparing it to initial vertical defect size (T6 – T8)<br><br>2. Regeneration rate / dividing the bone gain to the initial defect size (T6 – T8)<br><br>3. Bone quality / Histological and histomorphometric analyses (T6 – T8)<br><br>4. Surgical and healing complications / Clinical evaluations (T6 – T8)	1. 17 out of the 19 cases saw no type of surgical or healing complications and all had successful primary closures (the non-exposure group).<br><br>2. One of the cases saw late non-infectious exposure of titanium meshes that was resolved.<br><br>3. One of the cases saw early infectious exposure of titanium meshes that les to premature removal of the mesh and being excluded from receiving dental implants.<br><br>4. In 11 out of the cases, vertical bone gain was observed up to the height of adjacent bone.<br><br>5. Healing period:<br>5.1. All patients: 8.0 ± 1.4 months<br>5.2. Non-exposure group (n = 17): 8.0 ± 1.5 months<br>5.3. Exposure group (n = 2): 7.8 ± 0.7 months

Author (Year) / Study Type<br><br>***A) Number of Patients***<br>***B) Gender (M:F)***<br>***C) Age***	***A) Surgical Procedure***<br>***B) Graft Material***	***A) Study Phases***<br>***B) Study Groups***<br>***C) Dental Implants***<br>***D) Alveolar Defects***	***Study Variables / Evaluation Methods (Periods)***	***Outcomes and Complications***
		D)<br>D.1. Defect locations:<br>- Posterior mandible: 3<br>- Posterior maxilla: 4<br>- Anterior mandible: 2<br>- Anterior maxilla: 10<br><br>D.2. Mean defect size: 10.0 ± 3.8 mm (ranged from 2.3 mm to 15.0 mm)		6. Vertical bone gain:<br>6.1. All patients: 8.6 ± 4.0 mm<br>6.2. Non-exposure group (n = 17): 8.8 ± 4.2 mm<br>6.3. Exposure group (n = 2): 7.2 ± 0.2 mm<br><br>7. Regeneration rates:<br>7.1. All patients: 85.8% ± 25.5%<br>7.2. Non-exposure group (n = 17): 87.3% ± 25.6%<br>7.3. Exposure group (n = 2): 73.4% ± 37.7±<br><br><br>**<u>Conclusions:</u>**<br>Using cross-slinked collagen membranes on top of titanium meshes in vertical defects filled with bone graft and rhPDGF-BB results in substantial vertical bone gain. However, long-term evaluations were not included in this study.

Supplementary Table S2. Detailed quality assessment of the included randomized studies.

Domain	*Item*	*Cucchi et al. (2021 and 2024)*	*Cucchi et al. (2017, 2019, 2021 and 2023)*	*Urban et al. (2025)*
1. Randomization process	1.1<br>1.2<br>1.3<br>1.0 Assessor's judgment	Y<br>Y<br>PN<br>Low	Y<br>Y<br>PN<br>Low	Y<br>Y<br>PN<br>Low
2. Deviations from intended interventions	2.1<br>2.2<br>2.3<br>2.4<br>2.5<br>2.6<br>2.7<br>2.0 Assessor's judgment	PN<br>Y<br>PN<br>NA<br>NA<br>Y<br>NA<br>Low	PN<br>Y<br>PN<br>NA<br>NA<br>Y<br>NA<br>Low	PN<br>Y<br>PN<br>NA<br>NA<br>Y<br>NA<br>Low
3. Missing outcome data	3.1<br>3.2<br>3.3<br>3.4<br>3.0 Assessor's judgment	Y<br>NA<br>NA<br>NA<br>Low	PN<br>PN<br>PY<br>N<br>Some Concerns	Y<br>NA<br>NA<br>NA<br>Low
4. Measurement of the outcome	4.1<br>4.2<br>4.3<br>4.4<br>4.5<br>4.0 Assessor's judgment	PN<br>PN<br>N<br>NA<br>NA<br>Low	PN<br>PN<br>N<br>NA<br>NA<br>Low	PN<br>PN<br>N<br>NA<br>NA<br>Low
5. Selection of the reported result	5.1<br>5.2<br>5.3<br>5.0 Assessor's judgment	PY<br>PN<br>PN<br>Low	PY<br>PN<br>PN<br>Low	PY<br>PN<br>PN<br>Low
Overall judgement	-	Low	Some Concerns	Low

Note: The detailed questions for each domain are according to the "Revised Cochrane risk-of-bias tool for randomized trials (RoB 2) SHORT VERSION (CRIBSHEET)".

Supplementary Table S3. Detailed quality assessment of the included non-randomized studies.

Domain	***Item***	***Funato et al. (2013)***
1. Confounding	1.1<br>1.2<br>1.3<br>1.4<br>1.0 Assessor's judgment	Y<br>NA<br>NA<br>NA<br>Low
2. Classification of interventions	2.1<br>2.2<br>2.3<br>2.4<br>2.5<br>2.0 Assessor's judgment	PN<br>PN<br>Y<br>PN<br>PY<br>Low
3. Selection of participants	3.1<br>3.2<br>3.3<br>3.4<br>3.5<br>3.6<br>3.7<br>3.8<br>3.9<br>3.10<br>3.0 Assessor's judgment	PN<br>PN<br>PY<br>NA<br>PY<br>PY<br>PN<br>NA<br>NA<br>NA<br>Moderate
4. Deviations in intended interventions	4.1<br>4.2<br>4.3<br>4.4<br>4.5<br>4.0 Assessor's judgment	PN<br>NA<br>NA<br>NA<br>Y<br>Low
5. Missing data	5.1<br>5.2<br>5.3<br>5.4<br>5.5<br>5.6<br>5.7<br>5.8	PY<br>PY<br>PY<br>NA<br>NA<br>NA<br>NA<br>NA

Domain	***Item***	***Funato et al. (2013)***
	5.9<br>5.10<br>5.11<br>5.0 Assessor's judgment	NA<br>NA<br>NA<br>Low
6. Measurement of outcome	6.1<br>6.2<br>6.3<br>6.0 Assessor's judgement	N<br>PY<br>N<br>Low
7. Selection of the reported result	7.1<br>7.2<br>7.3<br>7.4<br>7.0 Assessor's judgement	PN<br>PN<br>PN<br>PN<br>Low
Overall judgement	-	Moderate

Note: The detailed questions for each domain are according to the "Cochrane risk-of-bias tool for non-randomized studies (ROBINS-I V2)".
